# Focused Ultrasound for Movement Disorders: A Bibliometric Analysis Revealing a Critical Research Gap in Mexico

**DOI:** 10.5334/tohm.1255

**Published:** 2026-10-30

**Authors:** Hector R. Martinez, Francisco A. Luna-Rangel, O. Gutierrez-Treviño, Cecilio Armengol-Garcia, Juan Pablo Santos-Martinez, Brenda Gonzalez-Bedolla, Jose A. Figueroa-Sanchez

**Affiliations:** 1 Tecnologico de Monterrey, Escuela de Medicina y Ciencias de la Salud, Monterrey, Nuevo Leon, Mexico; 2 Instituto de Neurologia y Neurocirugia, Centro Medico Zambrano Hellion, TecSalud, San Pedro Garza Garcia, Nuevo Leon, Mexico

**Keywords:** focused ultrasound, essential tremor, Parkinson’s disease, bibliometric analysis, health disparities, Mexico

## Abstract

**Background:**

Magnetic resonance-guided focused ultrasound (MRgFUS) thalamotomy is an established incisionless treatment for essential tremor and tremor-dominant Parkinson’s disease. The geographic distribution of research activity and Latin American participation remain incompletely characterized.

**Methods:**

We analyzed focused ultrasound publications in movement disorders indexed in Web of Science and Scopus from inception through September 22, 2026. Records were screened against prespecified eligibility criteria and analyzed with bibliometrix in R. We assessed publication trends, countries, authors, collaboration networks, and keywords. Country participation was examined by corresponding-author country and all-author affiliations. SciELO was searched separately to identify regional literature missed by the primary databases.

**Results:**

The final corpus included 1,907 documents from 546 sources and 5,503 authors. Annual publication growth was 8.56%, with output increasing from 55 publications in 2016 to 258 in 2024. Among 1,596 records with an assignable corresponding-author country, the United States accounted for 536 (33.6%), followed by China and Canada. No Latin American country ranked among the ten most productive. Forty-five publications included at least one Latin American affiliation, including 9 with a Mexican affiliation. None reported clinical outcomes from patients treated with focused ultrasound for a movement disorder in Mexico. SciELO identified one additional regional record but no Mexican clinical series.

**Discussion:**

Focused ultrasound research in movement disorders has expanded substantially but remains geographically concentrated. Latin American participation is emerging, but Mexico has yet to contribute published clinical outcome data. Prospective registries and multicenter regional collaborations could strengthen locally relevant evidence.

**Highlights:**

Global scientific production on focused ultrasound for movement disorders has grown steadily over the past decade, with a marked acceleration after the 2016 FDA approval for essential tremor, and remains concentrated in a small number of high-income countries. Clinical output has begun to appear in Latin America, with series reported from Chile and Argentina, but Mexico has adopted the technology without contributing clinical outcome data. Treatment decisions at the single Mexican center currently offering the procedure therefore rest on evidence generated in other populations.

## Introduction

Parkinson’s disease and essential tremor are among the most prevalent movement disorders worldwide, with their global burden increasing disproportionately over the past two decades [1,2]. In Mexico, epidemiological data remain scarce, and the true incidence and prevalence of both conditions are poorly characterized [3,4]. This gap in basic epidemiological knowledge reflects a broader pattern of underinvestment in movement disorders research within the country.

Until recently, deep brain stimulation (DBS) represented the primary device-assisted therapeutic option for patients with medically refractory tremor. However, access to DBS in Mexico remains limited and geographically uneven, with specialized centers concentrated in major urban areas. Reported barriers include high procedural costs, lack of local DBS centers or teams in many regions, prolonged travel distances or waiting times for patients, and insufficient physician training or referral pathways [5]. In 2016, magnetic resonance-guided focused ultrasound (MRgFUS) thalamotomy received approval from the United States Food and Drug Administration (FDA) for medication-refractory essential tremor, following the results of a pivotal randomized controlled trial [6,7]. Subsequent clinical evidence supported its use for medication-refractory tremor in tremor-dominant Parkinson’s disease [8], and the FDA expanded the indication to this population in 2018 [9]. Unlike DBS, MRgFUS does not require implanted hardware or a cranial incision, thereby avoiding hardware-related maintenance and some of the procedural requirements associated with implantable neuromodulation systems [10].

Despite this therapeutic promise, global scientific production on MRgFUS for movement disorders remains concentrated in high-income countries [11]. Bibliometric analysis provides a validated quantitative framework to map these scientific landscapes, identify productive research hubs, and expose gaps where output fails to reflect disease burden [12]. To our knowledge, no study has characterized FUS research in movement disorders through a health equity lens or systematically examined the underrepresentation of Latin American research in this field.

## Methods

### Study design

We conducted a bibliometric analysis of the global literature on focused ultrasound applied to movement disorders. The primary searches were performed in Web of Science and Scopus on September 22, 2026. Records from both databases were retrieved on the same day to minimize discrepancies related to database updates. A complementary search of SciELO was conducted to assess the potential underrepresentation of literature published in Latin America and in Spanish- and Portuguese-language sources.

### Data sources and search strategy

Web of Science and Scopus were selected as the primary bibliographic databases because of their broad coverage of peer-reviewed biomedical literature, availability of citation metadata, and compatibility with bibliometric analysis software. The search strategy combined focused ultrasound-related terms with terms related to movement disorders, ablative procedures, and relevant neuroanatomical targets. Focused ultrasound terms included “*focused ultrasound,” “focussed ultrasound,” “high intensity focused ultrasound,” “high-intensity focused ultrasound,” HIFU, MRgFUS, tcMRgFUS, “MR guided focused ultrasound,” “magnetic resonance guided focused ultrasound,” “transcranial focused ultrasound,” and Exablate. Movement disorder and procedure-related terms included Parkinson, “essential tremor,” tremor, “movement disorder,” dystonia, thalamotomy, subthalamotomy, pallidotomy, subthalamic, “globus pallidus,” and “ventral intermediate nucleus*.” Database-specific syntax, plural forms, and truncation operators were applied as appropriate for Scopus and Web of Science. The complete database-specific search strategies are provided in Supplementary Material 1.

The search strategy was initially developed in Scopus using the TITLE-ABS-KEY field and was subsequently adapted to the corresponding field syntax in Web of Science. No restrictions were applied by publication year, language, or document type, and both databases were searched from inception through September 22, 2026.

To assess whether the primary databases might have missed relevant regional literature, we performed an additional search in SciELO using equivalent focused ultrasound and movement disorder terms in English, Spanish, and Portuguese. SciELO records were assessed separately and cross-checked against the Web of Science and Scopus corpus by digital object identifier and publication title rather than incorporated into the primary bibliometric dataset.

The complete search strategies for Web of Science, Scopus, and SciELO, including the exact search strings, field tags, and language-specific terms, are provided in Supplementary Material 1.

### Eligibility criteria and study selection

Records were eligible when they met both of the following criteria. First, focused ultrasound had to represent a substantive component of the study. Eligible applications included magnetic resonance-guided focused ultrasound, transcranial focused ultrasound, high-intensity focused ultrasound, and low-intensity ultrasound neuromodulation, whether used for tissue ablation, neuromodulation, or blood-brain barrier opening. Technical and methodological studies addressing acoustic fields, transducer design, skull transmission, or acoustic windows were also eligible when these aspects were investigated in the context of focused ultrasound applications relevant to the field.

Second, the study had to address a movement disorder or a neurosurgical target directly relevant to movement disorders. Eligible conditions included essential tremor, Parkinson’s disease, dystonia, Huntington’s disease, chorea, ataxia, Tourette syndrome, tic disorders, myoclonus, and restless legs syndrome. Studies involving established or investigational surgical targets, including the ventral intermediate nucleus of the thalamus, subthalamic nucleus, globus pallidus, and substantia nigra, were also eligible. Preclinical, translational, and clinical studies were retained, with no restrictions according to language, publication year, or document type.

Records were excluded when focused ultrasound was mentioned only incidentally and was not substantively examined; when focused ultrasound was investigated for an indication outside the movement disorders field, such as oncology, pain, epilepsy, obsessive-compulsive disorder, depression, Alzheimer’s disease, or stroke, without a relevant movement disorder component; when the anatomical site of interest was outside the central nervous system; when ultrasound was used for drug or nanoparticle delivery as one of several delivery modalities without specific evaluation of its application to movement disorders; when the study evaluated diagnostic ultrasound, including transcranial sonography of the substantia nigra; or when the primary intervention was an alternative to focused ultrasound, such as deep brain stimulation, stereotactic radiosurgery, or laser interstitial thermal therapy, and the study neither reported focused ultrasound outcomes nor directly compared the alternative intervention with focused ultrasound. These criteria were applied during manual eligibility screening of all unique records.

Records retrieved from Web of Science and Scopus were combined and de-duplicated using digital object identifiers and, when no identifier was available, publication title and year. All unique records were independently screened by two reviewers against the prespecified eligibility criteria. Screening was based on titles and abstracts, with full-text assessment when the available bibliographic information was insufficient to determine eligibility. Disagreements were resolved through discussion, and unresolved cases were adjudicated by a third reviewer.

A rule-based classifier was applied to the complete set of records as an auxiliary screening tool to facilitate record prioritization. Classifier decisions were not used to determine eligibility or to exclude records from manual review. All unique records underwent independent assessment by both reviewers according to the prespecified eligibility criteria, and final inclusion decisions were based exclusively on human review. Agreement between the two reviewers was quantified using Cohen’s kappa, yielding κ = 0.86.

### Data extraction and processing

Full bibliographic records were exported from Web of Science and Scopus, including authors, institutional affiliations, source titles, document types, author keywords, Keywords Plus terms where available, citation counts, and cited references. Records were imported into R and converted into standardized bibliographic data frames using the bibliometrix package [13]. The two datasets were subsequently merged into a single bibliographic corpus, with duplicate records identified by digital object identifier and, when necessary, publication title and year.

Only records meeting the prespecified eligibility criteria were retained in the primary bibliometric corpus. Document type was not used as an eligibility criterion and was instead characterized descriptively in the final dataset.

SciELO records were evaluated separately using the same eligibility criteria and were manually cross-checked against the primary corpus using digital object identifiers and titles. The SciELO search was used as a regional sensitivity analysis and was not incorporated into the primary bibliometric calculations.

### Bibliometric analysis

The final eligible corpus was analyzed in R using the bibliometrix package. We summarized the principal characteristics of the dataset, including publication timespan, number of documents and sources, number of authors, annual growth rate, document age, average citations per document, document types, and international co-authorship.

Annual scientific production was examined across the study period. We identified the most productive authors and sources and the most frequent author keywords and Keywords Plus terms. Equivalent keyword variants were consolidated before co-occurrence analyses to reduce fragmentation arising from differences in spelling, capitalization, abbreviations, and terminology.

Geographic output was evaluated using two complementary approaches. First, countries were ranked according to the corresponding author’s country for records in which this information could be assigned. Percentages derived from this analysis were calculated using the number of records with an assignable corresponding-author country as the denominator. Second, participation by country was assessed using the affiliations of all authors, allowing identification of publications involving investigators from a country even when the corresponding author was based elsewhere. Latin American and Mexican participation were additionally quantified as the number of records containing at least one institutional affiliation from the respective region or country.

Records involving Mexican institutions were identified through the author affiliation fields and were reviewed individually to characterize study design, research focus, institutional participation, and whether clinical outcomes from patients treated in Mexico were reported.

International research collaboration was evaluated using a country-level co-authorship network generated with the biblioNetwork and networkPlot functions in bibliometrix. The network visualization included the 30 most prominent country nodes, used a Fruchterman layout, and applied Louvain community detection. Multiple edges and isolated nodes were removed for visualization.

### Software and reproducibility

All analyses were performed in R version 4.6.0 using bibliometrix version 5.4.1. Custom R scripts were used for database harmonization, duplicate identification, eligibility-based filtering, extraction of country information, descriptive analyses, and figure generation. The complete database search strategies, eligibility criteria, screening workflow and additional methodological details are provided in Supplementary Material 1.

## Results

### Overview of the corpus

The primary searches retrieved 1,598 records from Web of Science and 1,424 from Scopus, yielding 3,022 records before database merging. After removal of duplicate records, 2,012 unique records remained. Following eligibility-based filtering, the analyzed corpus comprised 1,907 documents published between 1960 and 2026 across 546 sources and authored by 5,503 researchers.

Articles were the most frequent document type (n = 947, 49.7%), followed by reviews (n = 410, 21.5%), meeting abstracts (n = 198, 10.4%), letters (n = 81, 4.2%), editorial material (n = 63, 3.3%), and book chapters (n = 49, 2.6%). The remaining 159 records (8.3%) comprised less frequent or composite document-type categories, including editorials, proceedings papers, notes, early-access records, conference papers, corrections, errata, conference reviews, short surveys, abstract reports, and news items. The complete distribution of document types is provided in Supplementary Table 1.

The annual growth rate of publications was 8.56%, the mean document age was 4.51 years, and documents received an average of 22.38 citations. Publications had an average of 6.8 co-authors, 118 documents were single-authored, and 19.72% involved international co-authorship.

### Temporal trend

Scientific output remained limited before 2010. Publication activity subsequently increased, from 55 documents in 2016 to a peak of 258 in 2024. A total of 243 documents were published in 2025, while the 226 records indexed for 2026 reflect a partial year because the search was conducted on September 22, 2026 (Figure 1).

**Figure 1:**
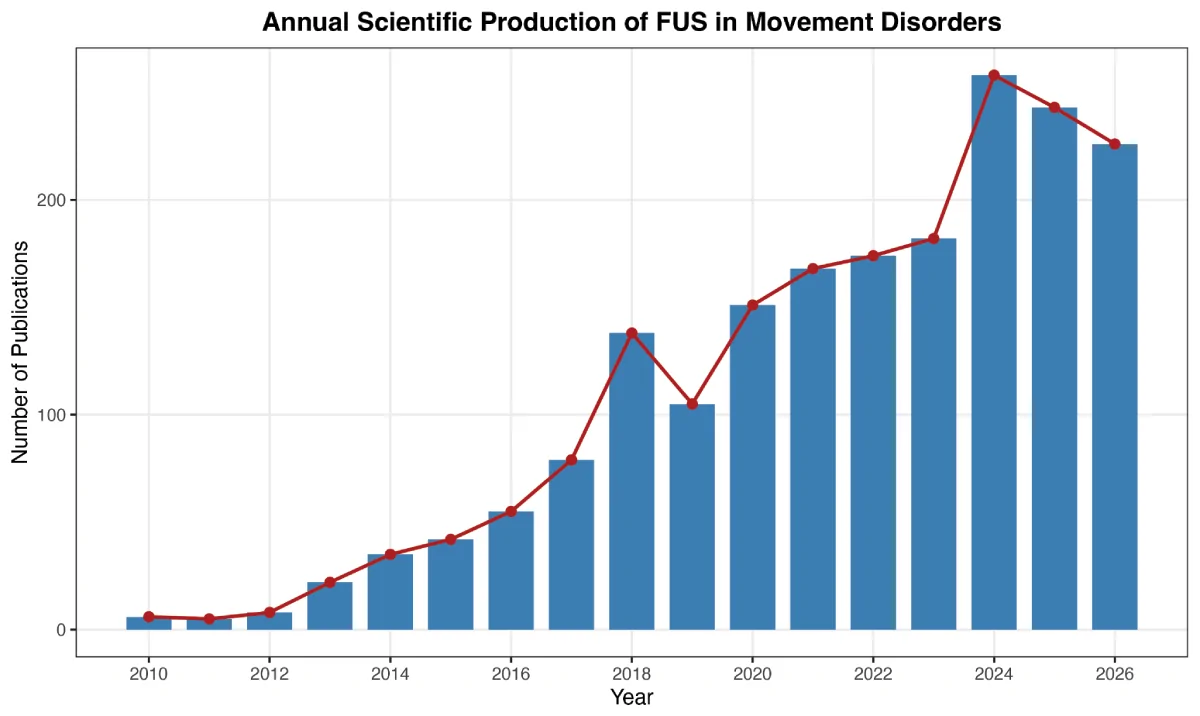
**Annual scientific production of focused ultrasound research in movement disorders (2010–2026)**. Records were retrieved from Web of Science and Scopus, searched on 22 September 2026. The red line indicates the publication trend. Annual output rose from 55 publications in 2016, the year of FDA approval of MRgFUS for essential tremor, to a peak of 258 in 2024. Only 10 documents in the corpus predate 2010, and the figure therefore begins in that year. Data for 2026 are incomplete, covering January to September.

### Leading authors

Andres Lozano was the most productive author, with 83 publications, followed by W. Jeffrey Elias (n = 67), José Obeso (n = 66), N. Lipsman (n = 61), and Juan Pineda-Pardo (n = 55).

### Sources and research themes

*Movement Disorders* was the most productive source, with 205 publications, followed by the *Journal of Neurosurgery* (n = 95), *Neurosurgery* (n = 55), *Frontiers in Neurology* (n = 47), and *Stereotactic and Functional Neurosurgery* (n = 43).

After consolidation of synonymous and closely related keyword variants, the most frequent author keywords were “essential tremor” (n = 397), “focused ultrasound” (n = 377), “Parkinson’s disease” (n = 363), “MR-guided focused ultrasound” (n = 307), and “thalamotomy” (n = 236). The keyword co-occurrence network and thematic map are presented in Supplementary Figures 1 and 2, respectively.

### Geographic distribution

Country information for the corresponding author could be assigned to 1,596 records. Among these, the United States accounted for 536 publications (33.6%), followed by China with 190 (11.9%) and Canada with 161 (10.1%). Italy and Spain each accounted for 85 publications (5.3%). No Latin American country ranked among the ten most productive countries by corresponding-author affiliation. Brazil was the highest-ranking Latin American country, with 13 publications, ranking fifteenth overall (Figure 2).

**Figure 2:**
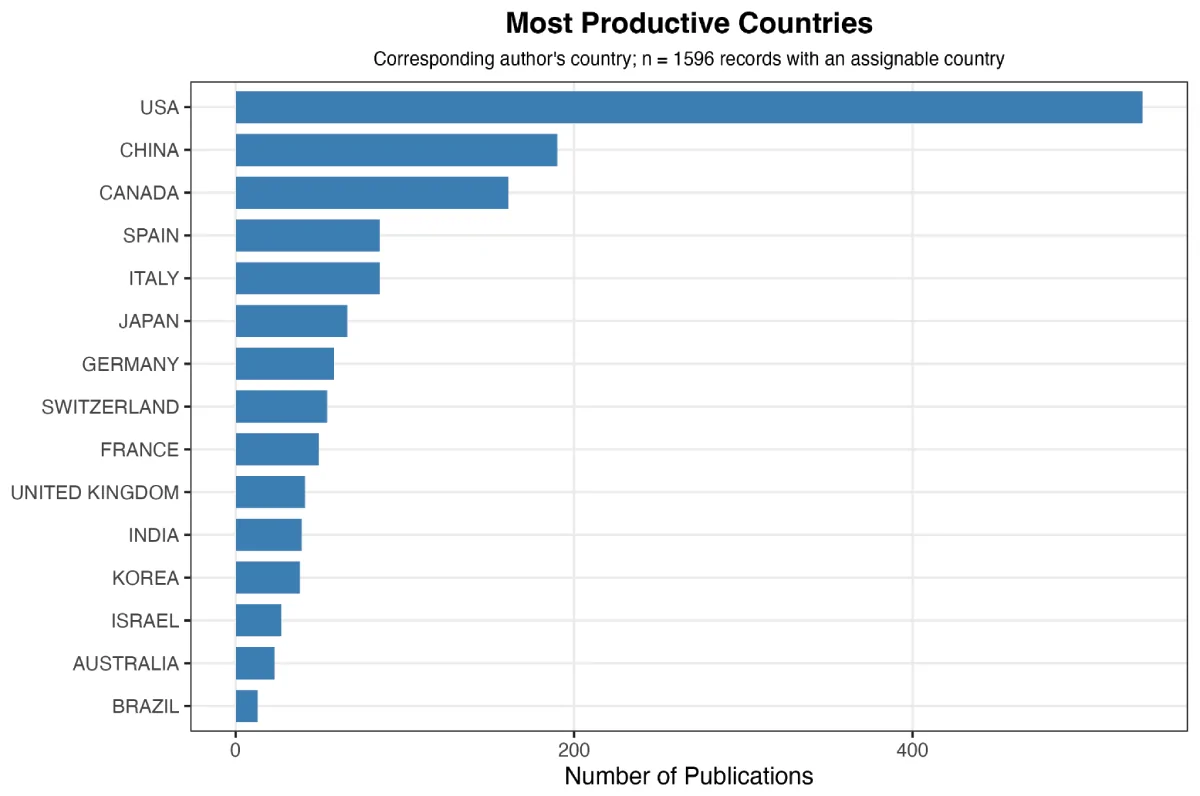
**Most productive countries in focused ultrasound research for movement disorders. Countries are ranked according to the corresponding author’s country of affiliation**. Percentages were calculated using the 1,596 records for which a corresponding-author country could be assigned, rather than the full corpus of 1,907 publications. The United States ranked first with 536 publications (33.6%), followed by China (n = 190) and Canada (n = 161). Brazil was the highest-ranking Latin American country, placing fifteenth with 13 publications. Mexico accounted for six publications when classified by corresponding-author country.

When geographic participation was assessed using the affiliations of all authors, the United States participated in 678 publications, followed by Canada in 222 and China in 221. Overall, 45 publications included at least one Latin American institutional affiliation, whereas 22 had a corresponding author based in the region.

### Output and collaboration involving Mexico

Nine publications included at least one Mexican institutional affiliation. These records were reviewed individually to characterize study design, institutional participation, and clinical relevance. None reported clinical outcomes from patients treated with focused ultrasound for a movement disorder in Mexico. Their characteristics are summarized in Table 1.

**Table 1:** Characterization of publications with Mexican institutional affiliation on focused ultrasound for movement disorders indexed in Scopus and Web of Science (inception through September 22, 2026). **BBB:** blood-brain barrier; **FUS:** focused ultrasound; **MRgFUS:** magnetic resonance-guided focused ultrasound; PD: Parkinson’s disease. **CINVESTAV:** Center for Research and Advanced Studies of the National Polytechnic Institute; **IMSS:** Mexican Social Security Institute; **INNN:** National Institute of Neurology and Neurosurgery; **INR:** National Rehabilitation Institute; **IPN:** National Polytechnic Institute; **UNAM:** National Autonomous University of Mexico.


FIRST AUTHOR AND YEAR	JOURNAL	STUDY TYPE	MAIN FOCUS OR OBJECTIVE	KEY FINDING RELEVANT TO MOVEMENT DISORDERS	MEXICAN INSTITUTIONAL AFFILIATION	CLINICAL OUTCOMES IN MEXICAN PATIENTS?

Alshimemeri (2022) [14]	Journal of Neurology, Neurosurgery & Psychiatry	Case Report	Describe functional tremor following MRgFUS thalamotomy for essential tremor.	Identifies the development of functional movement disorders as a potential post-operative complication	Yes (INNN)	No

Mascotte-Cruz (2024) [15]	Discover Nano	Preclinical Animal	Utilize FUS for targeted gene delivery to nigral dopaminergic neurons via nanoparticles	FUS safely facilitates targeted delivery to the substantia nigra, showing potential for PD gene therapy	Yes (CINVESTAV; UNAM; IPN; IMSS)	No

Gutierrez (2024) [16]	J. Phys. Mater.	Translational/Engineering	Characterize alumina ceramic disks for use as cranial “acoustic windows”	Resonant alumina disks allow 95.4% ultrasound transmission, potentially aiding chronic brain therapy	Yes (INR; CINVESTAV)	No

Valerio (2025) [17]	Brain Sciences	Review	Explore AI applications in neurosurgery for Parkinson’s disease	MRgFUS provides non-invasive tremor relief; AI can optimize target selection and outcomes	Yes (Tecnológico de Monterrey)	No

Rodríguez (2023) [18]	Pharmaceutics	Preclinical Animal	Animal Develop a protocol for temporary, reversible BBB opening in the substantia nigra	A 10 mm craniotomy allowed accurate US focalization; the BBB remained open for 6 hours post-FUS	Yes (CINVESTAV; INR) No	No

Rodríguez (2019) [19]	GMEPE/PAHCE	Preclinical Animal	Open the BBB in the substantia nigra using FUS and microbubbles in rats	Low power (3 W) combined with microbubbles effectively and reversibly opens the BBB	Yes (CINVESTAV; INR)	No

Hernández (2017) [20]	GMEPE/PAHCE	Translational/Modeling	Model acoustic fields to determine optimal craniotomy dimensions for FUS	A 5.1 mm craniotomy provides the highest pressure concentration without deforming the transducer focus	Yes (CINVESTAV; INR)	No

Gutierrez (2017) [21]	Adv. Healthcare Mater.	Translational/Engineering	Propose 8YSZ ceramics as permanent acoustic windows for brain therapy	Pore-free 8YSZ polycrystals act as effective acoustic windows, achieving 81% transmission efficiency	Yes (INR; CINVESTAV)	No

Contreras (2016) [22]	GMEPE/PAHCE	Translational/Modeling	Model HIFU acoustic fields for BBB opening in rat models of Parkinson’s	Finite element modeling provides thermal and radiation patterns to guide drug delivery experiments	Yes (CINVESTAV)	No


Mexico’s strongest international collaboration was with the United States, with four co-authored publications, followed by Canada, Spain, and Germany, with two each. Additional collaborations involving one publication each were identified with China, Italy, France, the United Kingdom, Israel, Australia, the Netherlands, Denmark, Austria, Saudi Arabia, Greece, and Uruguay (Figure 3).

**Figure 3:**
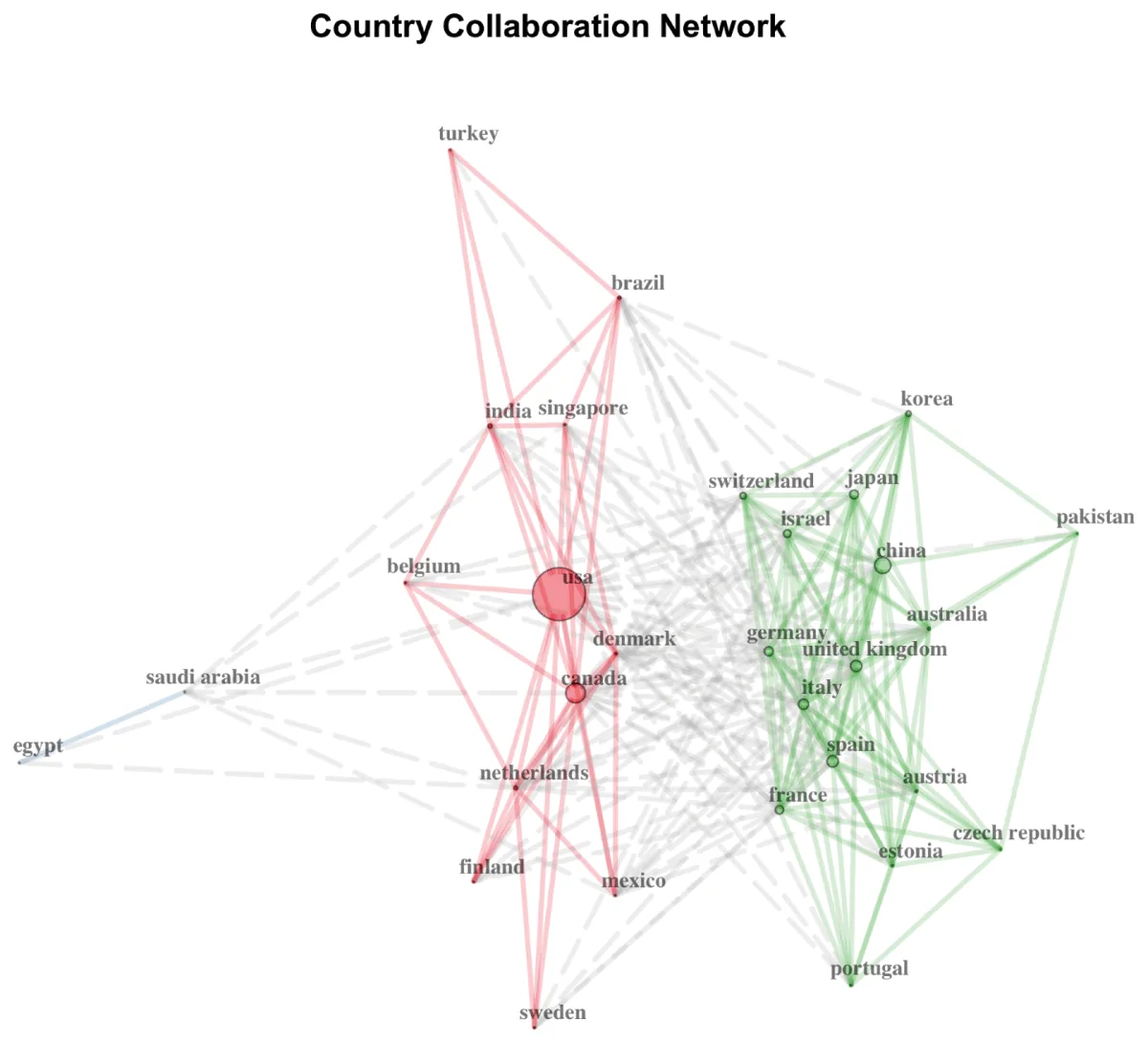
**International collaboration network in focused ultrasound research for movement disorders**. The network includes the 30 most prominent countries in the final bibliometric corpus. Node size reflects publication volume, edge thickness represents the strength of international co-authorship, and node colors indicate communities identified using Louvain clustering. The network was displayed using a Fruchterman layout. Mexico occupies a peripheral position, with its strongest collaborative link involving the United States, followed by Canada, Spain, and Germany.

### Regional sensitivity analysis

The complementary SciELO search retrieved six records. Five were already represented in the Web of Science and Scopus corpus. One additional Spanish-language review published in a Colombian journal was not retrieved by either primary database. No additional clinical series reporting focused ultrasound outcomes in Mexican patients were identified through the regional search.

## Discussion

The absence of published clinical outcome data on MRgFUS from Mexico has practical implications for local practice. Although MRgFUS thalamotomy has recently become available in the country, treatment decisions remain largely informed by evidence generated outside Mexico. The applicability of those findings to Mexican patients has not been examined directly, particularly in a setting where the epidemiology of Parkinson’s disease and other movement disorders remains incompletely characterized [3,4]. Prospective case series and clinical registries would provide an initial framework for evaluating outcomes in the local population.

Scientific output increased substantially over the study period, from 55 publications in 2016 to a peak of 258 in 2024. This increase coincided with major regulatory milestones, including FDA approval of MRgFUS thalamotomy for essential tremor in 2016 and expansion of the indication to tremor-dominant Parkinson’s disease in 2018 [6,9]. Regulatory approval may have contributed to broader clinical adoption and research activity, although the bibliometric design does not allow a causal relationship to be established. Other developments, including technical maturation of MRgFUS systems, expansion of clinical indications, and publication of pivotal clinical trials, occurred over the same period [7,8].

Research activity remains concentrated among a relatively small group of investigators and institutions, consistent with previous scientometric analyses of the field [11]. Andres Lozano was the most productive author in the present corpus, with 83 publications. The international collaboration network similarly showed a limited number of highly connected research hubs. Mexico occupied a peripheral position within this network, with its strongest collaborative link involving the United States, followed by Canada, Spain, and Germany. Despite these international connections, no publication identified in the present analysis reported clinical outcomes from patients treated with focused ultrasound for a movement disorder in Mexico.

Latin American participation was limited but not absent. Brazil had the largest regional publication output, while clinical reports have begun to emerge from other countries. Investigators in Chile have reported the predictive value of brain volumetry for outcomes after high-intensity focused ultrasound subthalamotomy in Parkinson’s disease and have described early regional experience with MRgFUS thalamotomy for tremor [23,24]. An Argentine center has also reported clinical outcomes in a series of 48 patients [25]. Clinical access to focused ultrasound in Latin America remains relatively recent, and the interval between clinical adoption and indexed publication may partly account for the limited regional output observed in this analysis. These reports indicate that clinical focused ultrasound research is beginning to emerge in the region, although indexed output remains limited. Mexico, in contrast, had no published clinical outcome series in the databases examined, and this finding was unchanged after extending the search to Spanish- and Portuguese-language sources.

The concentration of publications within a small number of journals further reflects the specialized nature of the field. Movement Disorders accounted for 205 publications and the Journal of Neurosurgery for 95, followed by a relatively small group of neurology, neurosurgery, and functional neurosurgery journals. This concentration parallels the geographic and institutional clustering observed in the authorship and collaboration analyses.

## Limitations

Several limitations should be considered. First, the primary bibliometric analysis was based on Web of Science and Scopus. These databases provide extensive citation metadata but may incompletely represent literature published in regional or non-English-language journals [26]. To examine this limitation, we conducted a complementary search in SciELO using terms in Spanish and Portuguese. Six records were identified, five of which were already represented in the primary corpus. One additional Spanish-language review published in a Colombian journal was identified, but no additional clinical series from Mexico were found.

Second, country-level productivity was primarily defined according to the corresponding author’s affiliation. This approach does not capture all international participation, particularly when investigators contribute to multinational studies without serving as corresponding authors. We therefore performed a complementary analysis based on the institutional affiliations of all authors. Mexican publications were similarly identified through affiliation metadata, which may be affected by inconsistencies in how institutional addresses are reported across bibliographic databases.

Third, citation-based measures reflect academic visibility and influence rather than methodological quality or clinical relevance [12]. Highly cited publications should therefore not be interpreted as necessarily providing stronger clinical evidence. Finally, bibliometric analyses capture published and indexed research rather than current clinical activity. Recently established clinical programs may therefore be underrepresented until their experience is published and indexed.

Focused ultrasound research for movement disorders has expanded substantially over the past decade but remains concentrated in a limited number of countries, institutions, and investigators. Latin American participation is emerging, with published clinical experience from Chile and Argentina, but remains modest relative to North America, Europe, and parts of Asia. Mexico has adopted the technology but has not yet contributed published clinical outcome data. Prospective registries, multicenter studies, and structured regional collaborations could help generate locally relevant evidence and improve representation of Latin American populations in the focused ultrasound literature.

## AI Disclosure

Grammarly and DeepL were used to assist with grammar correction and language editing during the preparation of this manuscript. All data processing, analysis, interpretation, and intellectual content are the sole responsibility of the authors.

## Additional File

The additional file for this article can be found as follows:

10.5334/tohm.1255.s1 Supplementary Material 1. Complete search strategies for Scopus, Web of Science, and SciELO; screening workflow and eligibility criteria; distribution of document types in the final bibliometric corpus (Supplementary Table 1); keyword co-occurrence network (Supplementary Figure 1); and thematic map (Supplementary Figure 2).
