# Supplementary material for: Focused Ultrasound for Movement Disorders: A Bibliometric Analysis Revealing a Critical Research Gap in Mexico
Source: Tremor Other Hyperkinet Mov (N Y). 2026 Oct 30;16:61. doi: 10.5334/tohm.1255 (PMC13722014; doi:10.5334/tohm.1255)

Search Strategy

Scopus

TITLE-ABS-KEY (

("focused ultrasound" OR "focussed ultrasound"

OR "high intensity focused ultrasound" OR "high-intensity focused ultrasound"

OR hifu OR mrgfus OR tcmrgfus

OR "MR guided focused ultrasound"

OR "magnetic resonance guided focused ultrasound"

OR "transcranial focused ultrasound"

OR exablate)

AND

(parkinson* OR "essential tremor" OR tremor OR tremors

OR "movement disorder" OR "movement disorders"

OR dystonia OR dystonias

OR thalamotomy OR subthalamotomy OR pallidotomy

OR subthalamic OR "globus pallidus" OR "ventral intermediate nucleus")

)

Web of Science

TS=(

("focused ultrasound" OR "focussed ultrasound"

OR "high intensity focused ultrasound" OR "high-intensity focused ultrasound"

OR HIFU OR MRgFUS OR tcMRgFUS

OR "MR guided focused ultrasound"

OR "magnetic resonance guided focused ultrasound"

OR "transcranial focused ultrasound"

OR Exablate)

AND

(Parkinson* OR "essential tremor" OR tremor*

OR "movement disorder*" OR dystoni*

OR thalamotom* OR subthalamotom* OR pallidotom*

OR subthalamic OR "globus pallidus" OR "ventral intermediate nucleus")

)

Scielo

("ultrasonido focalizado" OR "ultrasonido enfocado"

OR "ultrasonido focalizado de alta intensidad"

OR "ultrassom focalizado" OR "ultrassom focado"

OR "ultrassom focalizado de alta intensidade"

OR "focused ultrasound" OR HIFU OR MRgFUS)

AND

("enfermedad de Parkinson" OR "doenca de Parkinson" OR Parkinson

OR "temblor esencial" OR "tremor essencial" OR temblor OR tremor

OR "trastornos del movimiento" OR "disturbios do movimento"

OR distonia OR distonía

OR talamotomia OR talamotomía

OR subtalamotomia OR subtalamotomía

OR palidotomia OR palidotomía)

Screening workflow

The Web of Science search retrieved 1,598 records and the Scopus search retrieved 1,424 records, yielding 3,022 records before database merging. After removal of 1,010 duplicate records, 2,012 unique records remained. All unique records were independently screened by two reviewers according to the prespecified eligibility criteria. Screening was based on titles and abstracts, with full-text assessment when the available bibliographic information was insufficient to determine eligibility. Disagreements were resolved through discussion, with adjudication by a third reviewer when necessary. A total of 105 records were excluded for failing to meet the prespecified eligibility criteria, resulting in a final bibliometric corpus of 1,907 records. Agreement between the two reviewers was quantified using Cohen's kappa ($\kappa = 0.86$).

- 3,022 records retrieved
- 1,010 duplicates removed
- 2,012 unique records screened
- 105 records excluded
- 1,907 records included in the final corpus

Eligibility Criteria

Records were assessed against the criteria below, which were defined before screening began and applied unchanged throughout. The same criteria were used for records retrieved from

Web of Science and Scopus and for those retrieved from SciELO in the regional sensitivity analysis.

Inclusion criteria

A record was eligible only if it satisfied both of the following.

1. Focused ultrasound as a substantive component of the study.Eligible applications included magnetic resonance-guided focused ultrasound, transcranial focused ultrasound, high-intensity focused ultrasound, and low-intensity ultrasound neuromodulation, used for tissue ablation, neuromodulation, or blood-brain barrier opening. Technical and methodological work on acoustic fields, transducer design, skull transmission, or acoustic windows was eligible when these questions were addressed in the context of focused ultrasound applications relevant to the field.
2. A movement disorder, or a neurosurgical target directly relevant to movement disorders, as the clinical subject. Eligible conditions were essential tremor, Parkinson's disease, dystonia, Huntington's disease, chorea, ataxia, Tourette syndrome, tic disorders, myoclonus, and restless legs syndrome. Studies of established or investigational surgical targets were also eligible, specifically the ventral intermediate nucleus of the thalamus, the subthalamic nucleus, the globus pallidus, and the substantia nigra.
3. Preclinical, translational, and clinical studies were all retained. No restrictions were applied by language, publication year, or document type. Document type was recorded descriptively but was never used as an eligibility criterion.

Exclusion criteria

A record was excluded if any of the following applied.

1. Focused ultrasound was mentioned only incidentally and was not substantively examined.
2. Focused ultrasound was investigated for an indication outside the movement disorders field, such as oncology, pain, epilepsy, obsessive-compulsive disorder, depression, Alzheimer's disease, or stroke, without a relevant movement disorder component.
3. The anatomical site of interest lay outside the central nervous system.
4. Ultrasound was used for drug or nanoparticle delivery as one of several delivery modalities, without specific evaluation of its application to movement disorders.
5. The study evaluated diagnostic ultrasound, including transcranial sonography of the substantia nigra.
6. The primary intervention was an alternative to focused ultrasound, such as deep brain stimulation, stereotactic radiosurgery, or laser interstitial thermal therapy, and the study neither reported focused ultrasound outcomes nor directly compared the alternative intervention with focused ultrasound.

Supplementary Table 1. Distribution of document-type labels in the final bibliometric corpus (n = 1,907).

Document type	n	%
Article	947	49.7
Review	410	21.5
Meeting abstract	198	10.4
Letter	81	4.2
Editorial material	63	3.3
Book chapter	49	2.6
Editorial	29	1.5
Proceedings paper	27	1.4
Note	20	1.0
Article; early access	19	1.0
Conference paper	15	0.8
Review; early access	11	0.6
Correction	8	0.4
Article; proceedings paper	6	0.3
Article; book chapter	5	0.3
Letter; early access	4	0.2
Editorial material; early access	3	0.2
Erratum	3	0.2
Conference review	2	0.1
Review; book chapter	2	0.1
Short survey	2	0.1
Abstract report	1	0.1
Editorial material; book chapter	1	0.1
News item	1	0.1
**Total**	**1,907**	**100.0**

Supplementary Figure 1 Keyword co-occurrence network of focused ultrasound research in movement disorders. The 40 most frequent author keywords are shown; node size reflects keyword frequency and edge width reflects co-occurrence strength. Walktrap community detection identified three major clusters. The largest clinical cluster includes terms related to movement disorders and ablative procedures, including “essential tremor,” “Parkinson’s disease,” “thalamotomy,” “MRgFUS,” “pallidotomy,” and “deep brain stimulation.” A second cluster is centered on “focused ultrasound” and includes terms related to imaging, neuromodulation, thermal ablation, neurosurgery, and ultrasound terminology. A third cluster comprises preclinical and translational applications, characterized by terms such as “blood-brain barrier,” “drug delivery,” “microbubbles,” and “Alzheimer’s disease.” Connections between clusters indicate substantial overlap between clinical, technical, and translational areas of focused ultrasound research.

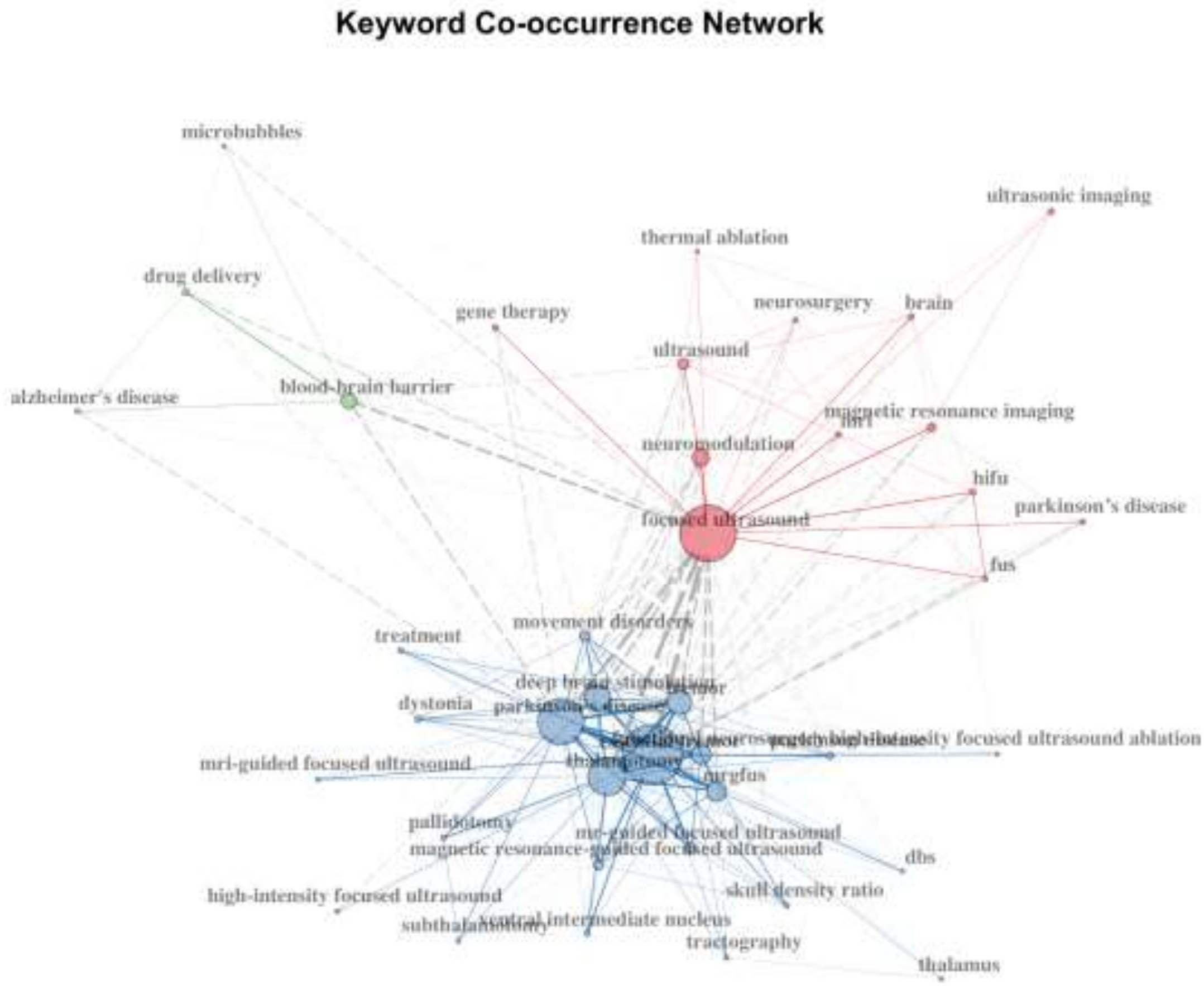

Supplementary Figure 2. Thematic map of focused ultrasound research in movement disorders. Themes are positioned according to Callon centrality (relevance degree) and Callon density (development degree), with dashed lines indicating the median values of each measure; bubble size reflects theme frequency. Five themes were identified. "Essential tremor" and "Parkinson's disease" are located within the Basic Themes quadrant, indicating high centrality but comparatively low density. "Safety" appears in the Niche Themes quadrant, reflecting a highly developed but relatively peripheral topic. "Ultrasound" lies near the median centrality boundary and above the median density threshold, whereas "focused ultrasound" lies near the median density boundary with comparatively lower centrality. No

theme is clearly positioned within the Motor Themes quadrant.

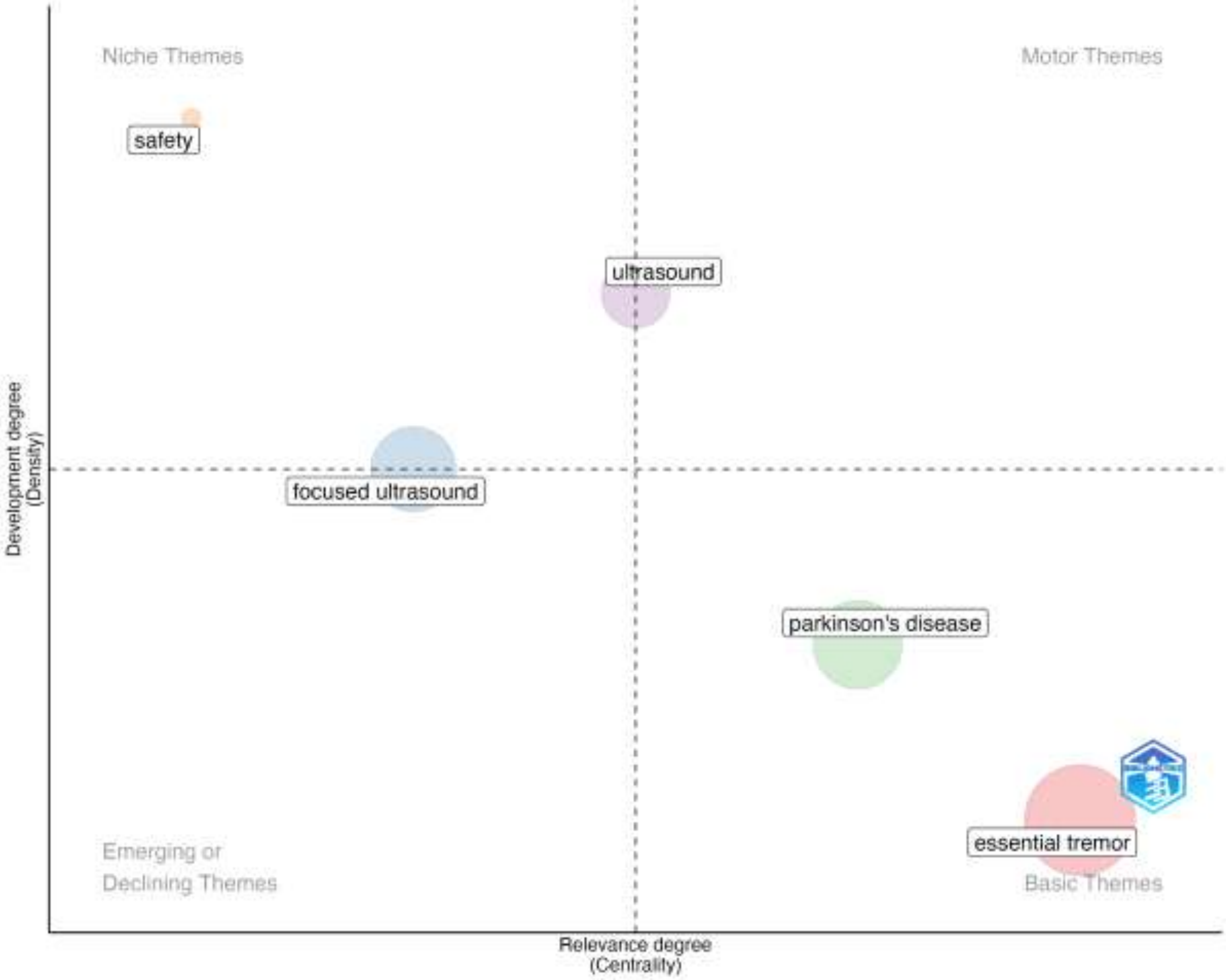

Supplement: Supplementary file 1 [file tohm-16-1-1255-s1.pdf]
